# Comparison of the validity of bookmark and Angoff standard setting methods in medical performance tests

**DOI:** 10.1186/s12909-020-02436-3

**Published:** 2021-01-02

**Authors:** Majid Yousefi Afrashteh

**Affiliations:** Department of Psychology, Faculty of Humanities, University of Zanjan, Zanjan, Iran

**Keywords:** Standard setting, Angoff, Bookmark

## Abstract

**Background:**

One of the main processes of determining the ability level at which a student should pass an assessment is standard setting. The current study aimed to compare the validity of Angoff and bookmark methods in standard-setting.

**Method:**

190 individuals with an M.Sc. degree in laboratory science participated in the study. A test with 32 items, designed by a group of experts, was used to assess the laboratory skills of the participants. Moreover, two groups each containing 12 content specialists in laboratory sciences, voluntarily participated in the application of the Angoff and bookmark methods. To assess the process validity, a 5-item questionnaire was asked from two groups of panelists. To investigate the internal validity, the classification agreement was calculated using the kappa and Fleiss’s Kappa coefficient. External validity was assessed by using five indices (correlation with criterion score, specificity, sensitivity, and positive and negative predictive values of correlation test with criterion score).

**Results:**

The results showed that the obtained cut-scores was 17.67 for Angoff and 18.8 for bookmark. The average total of items related to the quality of the execution process was 4.25 for the Angoff group and 4.79 for the bookmark group. Pass rates pass rates percentages for the Angoff and bookmark group were 55.78 and 41.36, respectively. Correlations of passing/failing, between employer ratings and test scores were 0.69 and 0.88 for Angoff and bookmark methods, respectively.

**Conclusion:**

Based on the results, it can be concluded that the process and internal validities of the bookmark method were higher than the Angoff method. For evaluation of the external validity (concordance of the cut score with the criterion score), all five external validity indices supported the bookmark method.

## Background

Student performance assessment is an important part of educational programs. Since learning objectives for university students set through performance assessment, higher education instructors and planners pay considerable attention to this issue [1, 2]. Educational systems try to level up the performance of learners to achieve predefined goals [3]. Setting the passing/failing grades and/or acceptable performance level (or minimum pass level) is the natural and common outcome of tests, which is important not only for the learners, but also for higher levels, including the school, city, state, and country [4]. Nevertheless, less attention is paid to setting the cut-off point or passing criterion [2].

Passing standard, passing criterion or minimum pass level are hypothetical boundary within the score range of a test that distinguishes individuals who have achieved mastery level from those who have not [5–7]. Standard-setting methods are used to determine the cut-score or minimum pass level [8, 9]. Typically, a fixed score, such as 10 or 12 out of 20, is considered to be the minimum pass level in educational examination at the university level and employment tests [10]. The use of a fixed pass level for all conditions is not fair due to the effect of such factors as the difference in item difficulty, execution of the test, level of subjects, and objective of the test. Therefore, educational justice is obtained when the minimum pass level in each test is set based on the conditions of that test [11].

In general, standard-setting methods are either item-centered or person-centered [9]. In item-centered methods (e.g., Angoff method), test content is reviewed by a group of experts and judges, whereas, in person-centered methods (e.g., borderline groups), the decision of judges is based on the actual performance of the subjects [8]. According to literature, Angoff [12] and bookmark [13] are two of the most common item-centered methods. The Angoff method has been proven by most of the evidence as the most common and best-known standard-setting method [14–16]. In the Angoff method, prior to the conduct of the test, a group of experts and judges are asked to review the content of each test question and then predict the likelihood of correct answer to each item by the borderline candidate. Then, the experts had the opportunity to discuss, to be aware of each other’s estimates, and to change their estimates if necessary. Finally, the mean of scores given by the judges to all questions is set as the passing standard and cut-score [16]. Nevertheless, this method is associated with difficulties, such as having a long-term procedure and need for an expert group [14, 17]. In addition, ambiguity in the concept of borderline candidate is among other limitations of this method [7, 14]. In an attempt to overcome the shortcomings of the Angoff method, researchers proposed a new method which, in addition to being suitable for both multiple-choice and constructed-response questions, reduces the experts’ work load, facilitates their decision-making, combines the experts’ decisions with measurement models in determining the cut-score, and considers the test content together with the performance level [18]. This method, named bookmark, was introduced by Mitzel, Lewis, Patz, and Green [13] and soon welcomed. In this method, the place of each item in the scale is first determined according to difficulty index extracted by item response theory (IRT), and then the items are placed in separate sheets from the easiest one to the most difficult item. The expert panel is then asked to place its bookmark somewhere between the questions, where they believe the probability of giving correct answer by the borderline candidate is 50% or 67% (10 or 13 of 20 possible points). Then, the average difficulty of the items identified by the judges was calculated. The cut-score in this method is determined by converting the mean ability score into raw scores. In the second round, data such as passing and failing rates, based on the obtained cut-score, is given to the experts. Based on the feedback, the experts are able to change their bookmarks between the items. In case of any change, the cut-score is determined again and is given to the panel. This process continues until a general consensus over cut-score is achieved [8, 19, 20].

Several studies compared the Angoff and bookmark approaches. Hsieh (2013) used Angoff and bookmark approaches to assess language proficiency levels of students [21]. To determine the three final cut-scores, they obtained the opinions of 32 experts and reported that using these two methods resulted in various mean scores. In this study, the strengths and weaknesses of each method were addressed. Buckendahl, Smith, Impara, and Plake [22] compared the Angoff and bookmark methods in standard-setting for a math test with 69 items. They used the opinions of a group of 23 experts. Both methods resulted in similar cut-scores. However, the standard deviation of the bookmark method was lower. Reckase compared the modified Angoff method and bookmark method with simulated data in an ideal condition, without any judgment and parameter estimation errors [23], and reported a negative bias in the bookmark method (i.e. the estimated cut-score was lower than the cut-score hypothesized by the experts). This study also showed that the modified Angoff method had a slight bias or was not skewed. Olsen and Smith (2008) compared the modified Angoff and bookmark methods for a home inspection certification and reported fairly similar results [24]. The results were also similar in terms of the standard error of judges and the initial cut-score. Schultz (2006) also reported similar results [25].

Since the implementation of all methods for standard-setting and selecting the best cut-score is not feasible, adopting an appropriate method is an important part of test construction. Hambleton and Pitoniak reviewed different comparative studies, but didn’t find any effective and generalizable result [26]. They emphasized the need for further comparative studies. Plake emphasized that the majority of literature support applying a specific method, and many factors, such as validity, should be examined [27]. Cizek and Bunch mentioned to current methodological problems of standard-setting methods [8]. According to Cizek, through investigation and comparison of the validity of different methods, the best one can be identified [28]. Kane (1994) emphasized the collection of evidence for evaluating three types of validity, namely process, internal, and external, to measure the validity of standard-setting methods. Process validity pertains to the accuracy of the execution process and trust of passing and performance grades, the internal validity is about the degree of agreement between experts, and external validity refers to the consistency of the estimated cut-score with an external objective criterion [5].

Given the importance of standard-setting methods in determining the passing grade, particularly in performance tests, the current study intended to compare the two common standard-setting methods, Angoff and bookmark, through comparing their validity indices. Previous comparisons of these two methods and other standard-setting methods were mainly theoretical or only compared the passing rates and standard errors; whereas, the current study aimed to compare the process, internal, and external validities of these two methods to reveal the advantages of each method based on validity indices.

## Method

190 individuals with an M.Sc. degree in laboratory science participated in the study, that were randomly selected from 30 active laboratories in Tehran. 55% were men, and the mean age of participants was 23.10 years. The most important inclusion criteria were a maximum of 3 months since graduation and at least 2 months of work experience. Besides, all participants had the experience of working in all parts of the lab.

A pencil-paper test, which had 32 multiple-choice questions, was used to assess the laboratory skills of participants. The items were designed by three laboratory supervisors and were reviewed, corrected, and approved by the three faculty members of the laboratory sciences. The questions were designed in a manner that their content was situational, and to cover various laboratory activities, including acceptance, sampling, device recognition, quality control, and analysis of laboratory tests’ results and reports. For example, a laboratory result test was given to the participants, and they were asked to distinguish between possible and close respaces.

Two groups each containing 12 content specialists in laboratory sciences, voluntarily participated in the application of the Angoff and bookmark methods. The inclusion criteria were being experienced in analyzing test items at least for 3 years and 5 years of experience in teaching students of laboratory sciences. To balance the panels, they represented various specializations in the field of laboratory sciences. This improved the understanding of the items and abilities of the experts. 60% of the participants were men. 35 of the specialists were experts in clinical biochemistry, 30% in hematology, 20% in immunology, and 30% in mycology. As specified by Chong, Taylor, Haywood [29] and Yudkowsky, Downing, Popescu [30] the members of the two panels were equal. Twenty-four specialists were randomly assigned to one of two groups, and it was ensured that the groups were equivalent in terms of age, gender, teaching background, and specialty of the members. To observe ethical considerations, participants were informed about the purpose of the research and the participation was voluntary and anonymous. One of the bookmark panelists in the last phase of the research did not participate due to illness, therefore, all related data were discarded. Hence, the results of the study were obtained from two groups, one with 11 members and the other with 12 members.

For Angoff’s method, a workshop was set up to introduce the executive process to the panelists. In the next session, test items were given to them to estimate the likelihood of answers of borderline candidate to each item. Then all items were reviewed by panelists and a likelihood score was assigned to each item individually. After individual decision making, panelists modified their decisions in two other rounds. In each round, the assigned likelihood scores were discussed to see each other’s estimates and change theirs. The results of the panelist discussions were recorded on a designed form. The mean score given by the panelists in the final round was set as the cut-score.

In the bookmark method, first, the training related to the bookmark implementation process was presented to the participant experts. Then, the items were calibrated, based on the item response theory (IRT), and ordered based on the difficulty parameter. Next panelists were asked to place their bookmarks somewhere between the questions, where they believed the likelihood of giving a correct answer by a borderline candidate is 50%. After determining items by the panelists, the difficulty levels of all judges were extracted, and the mean is calculated. The cut-score was determined by converting the obtained average difficulty scores to the corresponding ability scores and then determining its equivalent raw scores. In the second round, information such as passing and failing rates, based on the obtained cut-score, was given to the panelists. Based on the feedbacks, the experts were able to change the position of their bookmarks. The new cut-point in this stage was determined and given to the panelists. The same process was repeated in the third round. After confirmation of all panelists, the final cut-score was set.

To investigate the process validity of the two methods, a 5-item questionnaire was administered to measure the satisfaction of execution accuracy of each method. The face and content validities of the questionnaire were confirmed by the experts. To examine the internal validity, intra and inter agreement were considered using the Kappa and Fleiss’ Kappa coefficient. Besides, separate analyses were conducted for both methods. Intra-rater agreement referred to the correspondence of passing and failing rates according to the standards set by a panelist in the second, and final rounds of a method. The inter-panelist was defined as the agreement in the passing and failing rates between panelists at each rating stage of a method. To measure the external validity, in addition to the candidate’s score, the evaluation of the employer or supervisor of the candidate performance was also considered. To do so, eight indicators about the subject’s skills in the laboratory with zero (reject) and one (confirm) answers were provided to the evaluators. The final scores were between zero and eight. Employers were aware that the Total score of above 4 as acceptable and 4 and less than 4 were considered as rejected. This step aimed to investigate the degree of match between passing and failing rates with an external criterion in passing or failing the candidates. The reliability of this checklist was assessed using the Cronbach alpha coefficient, which was 0.76, indicating a good value. To investigate the external validity, five indices (correlation with criterion score, specificity, sensitivity, and positive and negative predictive values) were used. The tetrachoric correlation coefficient was used to investigate the association between passing/failing grades of the test with the criterion score (employer’s assessment). Sensitivity was defined as the probability of an assessment instrument detecting the the correct diagnosis of an individual who, according to the employer, has the target condition (in here, passing). Specificity was defined as the probability of an assessment instrument detecting the correct diagnosis of individuals who do not have the target condition (in here, failing). The positive predictive value of a test indicates the probability that a person who really has the condition will gain a positive test result (in here, passing). The negative predictive value of a test indicates the probability that a person who really does not have the condition will gain a negative test result (in here failing) [31].

## Results

The results include process validity, passing rate, internal validity, and external validity as follows.

To evaluate the process validity, the panelists were asked to give their opinions about five items. As shown in Table 1, only for the first item, the satisfaction from the Angoff method was greater. The highest difference was for the accuracy of methods, so that the mean score of The Angoff method and bookmark method was 3.88 and 5, respectively. Besides, the general mean values of the execution process were 4.25 for the Angoff method and the 4.79 for the bookmark group. The average of both groups was good, but the results of the independent t-test showed that the total mean in the bookmark group was significantly higher than the Angoff group (t = 2.74, df = 21 *p* = 0.01).

**Table 1:** Mean and standard deviation for proses validity indicators of two methods

Evaluation Items	Angoff		bookmark	
	M	SD	M	SD
1-Is the procedure was well understood?	4.66	0.44	4.52	0.89
2-Is the process of determining the cut score in this case was reasonable and appropriate?	4.13	0.59	4.88	0.40
3-Is working in the panel for members was desirable?	4.50	0.65	4.61	0.58
4-Is the overall process of this method was accurate?	3.88	0.71	5.00	0.00
5-After knowing the cut-off point, Is it true From your point of view?	4.04	0.50	4.89	0.33
Total	4.25	0.60	4.79	0.29

Cut Score and Pass Rate of the Angoff and bookmark Methods are presented in Table 2. As shown in the table, according to the final decisions, cut scores of the Angoff method and bookmark method are 17.67 and 18.82, respectively. The passing rate was 55.78% (*n* = 106) when the Angoff method was applied and 46.31% (*n* = 88) for the bookmark method. To compare frequencies, the chi-Square test was used and the results indicated no significant difference (chi square = 1.76, df = 1, *p* = 0.20). Although the differences were not significant, according to descriptive information, acceptance of the bookmark method was more. However, Angoff process had been understood better than the book process.

**Table 2:** Cut Score and Pass Rate of the Angoff and bookmark Methods

Methods	round	Cut score	Pass rate (%)
Angoff	1st	17.10	62.40
	2nd	17.50	58.12
	Final	17.67	55.78
bookmark	1st	18.40	50.50
	2nd	18.70	48.70
	Final	18.82	46.31

To examine the internal validity, the classification agreement was considered using the Kappa coefficient. Separate analyses were conducted for both groups. As shown in Table 3, in general, the Kappa agreement coefficient is higher in the bookmark method. To compare the intra-panelist degree of agreement in the two methods, the average agreement between the second and final rounds was calculated. The resulted average was 0.71 for the Angoff method and 0.94 for the bookmark method. To compare the two means, the result of the independent t-test was significant (t = 6.44, df = 21, *p* < 0.001), meaning that the intra-panelist degree of agreement in the bookmark panel was higher. Also, the agreement among the panelists was higher for the bookmark method than the Angoff method. The Fleiss’ Kappa coefficient was calculated to examine the inter-panelist degree of agreement. Fleiss’ Kappa for Angoff panel was 0.61 and 0.85 for the bookmark, both o were significant. According to Landis and Koch [32], to interpret the Kappa values, values from 0.61 to 0.80 indicate substantial agreement and 0.81 to 1.0 indicate almost perfect or perfect agreement. Meanwhile, Krippendorff [33] suggested that conclusions should be discounted for variables with values less than 0.67, tentative conclusions were made for values between 0.67 and 0.80, and definite conclusions were made for values above 0.80. Both intra-rater and inter-rater were higher in the bookmark method than the Angaf method.

**Table 3:** Intra-Panelist κ Coefficient Values for Each Method

Rater	Round	Angoff		bookmark	
		1st	2nd	1st	2nd
r1	2nd	0.64		0.70	
	Final	0.70	0.75	0.80	0.90
r2	2nd	0.69		0.80	
	Final	0.70	0.74	0.90	1.00
r3	2nd	0.70		0.79	
	Final	0.74	0.79	0.84	0.96
r4	2nd	0.78		0.85	
	Final	0.85	0.88	0.78	0.80
r5	2nd	0.75		0.80	
	Final	0.78	0.81	0.88	0.95
r6	2nd	0.76		0.84	
	Final	0.76	0.83	0.89	0.90
r7	2nd	0.64		0.81	
	Final	0.61	0.65	0.89	1.00
r8	2nd	0.48		0.85	
	Final	0.52	0.60	0.92	1.00
r9	2nd	0.49		0.90	
	Final	0.57	0.58	1.000	1.00
r10	2nd	0.45		0.87	
	Final	0.48	0.60	0.77	0.90
r11	2nd	0.60		0.80	
	Final	0.63	0.67	1.00	0.97
r12	2nd	0.47		–	
	Final	0.49	0.60	–	–

As shown in Table 4, the correlation between passing/failing, according to the employer, and passing/failing, according to the test score, was 0.69 in the Angoff method and 0.88 in the bookmark method. In both methods, the correlation coefficient at the alpha < 0.05 was significant. To test the difference between the two correlation coefficients after converting to z-score using Fisher’s r Steiger’s [34] Equations and became significant (z = 9.12, *p* < 0.001), which means that the decision made by the bookmark method is closer to the employer’s judgment. The other indices also showed that the passing/failing grades in the bookmark method were practically closer to the real passing/failing level set by the employer. In other words, the standard set by the bookmark method was more consistent with the actual performance of the subjects.

**Table 4:** External validity evaluation indicators for Angoff and bookmark methods

Method	External validity evaluation indicators				
	correlation	spe	sensi	Pos.pre	Neg.pre
Angoff	0.69	0.86	0.81	0.85	0.83
bookmark	0.88	0.92	0.96	0.90	0.98

*spe* Specificity, *sensi* Sensitivity, *pos.pre* Positive predictive, *Neg.pre* negative predictive

## Discussion

The standard setting is a key issue in performance tests. The current study aimed to perform a comprehensive comparison of two standard-setting methods for the laboratory skills test.

Angoff and bookmark methods were compared in terms of process, internal, and external validities as well as the achievement rate. The process validity of the two methods was compared using five items that were evaluated by the experts. It was found that the mean of validity indices was higher for the bookmark method; whereas, the mean of a better understanding of the procedure was higher for the Angoff method, which supports the better implementation process in the bookmark method. In a study conducted by Yim [35], despite minor differences in some indicators, no general difference was observed. It worth noting that this study used no statistical test. In the current study, the results of the independent t-test showed a significant difference between the mean of the two groups. Of course, the overall mean of both methods was evaluated at a desirable level. Given that before conducting the study, a workshop was held for participating experts, we had the same expectations for each of the two panels.

Intra and inter agreements were investigated using the Kappa and Fleiss’ Kappa coefficients to evaluate the internal consistency. Comparing the Angoff and bookmark methods showed that the panelists in the bookmark method had greater uniformity, which is consistent with the findings of Buckendahl, Smith, Impara, and Plake [22]. Although the study conducted by Yim [35] on the bookmark inter-rater agreement was better than that of Angoff, the intra-rater agreement on Angoff’s method is better reported. The results of the current study provide further evidence about the validity of the bookmark method in medical education performance-based assessments [36–38].

To evaluate the external validity (i.e., agreement of the cut-off score set in the test with the external criterion), the assessments made by the employers were used as Comparison criteria. All five external validity indices supported the bookmark method. The pass/fail scores obtained from the bookmark method were more similar to the judgment of the employers of the subjects. Olson and Smith [24] and Schultz [25] compared the bookmark and Angoff methods and reported a similarity concerning the standard error of the judges and cut-off scores. In most of the studies, the validity criteria, particularly the external validity, were not investigated. Meanwhile, some studies, including the study conducted by Yim [35], external validity is evaluated through comparing the results of other standard-setting procedures. Although this is common and confirmed in the sources [39, 40], but when intended to compare the external validity of two standard-setting procedures, it is better to consider an external criterion [41], due to implementation problems, but it’s less commonly used [40]. This is closer to Kane’s definition of external validity [5].

Based on the recommendation on validation methods provided by the Kane’s [5], the current study investigated three types of validities (i.e., process, internal, and external) that allowed for a more comprehensive comparison of the two methods. In general, the results of the current study support the superiority of the bookmark method over the Method of Angoff. In addition to the validity indices, the acceptance rates of the two methods were also compared. The lower achievement rate in the bookmark method implied the greater difficulty in reaching passing grade set by this method, that is in line with findings of the Çetin and Gelbal [4] and park [42]. This result may be useful for different purposes of performing the tests. Since external validity indices of the bookmark method were better, this strictness can be considered desirable.

The higher validity of the bookmark Method can be attributed to several factors. The bookmark method, which was developed to address the weaknesses of the Angoff method, combined the expert’s decisions with the advanced measurement models (IRT). More importantly, its focus is on the content of the test as well as the performance level. Also, since medical tests have clear practical content, in that the content and practice are very close, the cut-off point set by the bookmark method was more precise and consistent. Moreover, the bookmark method had a higher external validity than the Angoff method.

This study had several drawbacks.

a The test that was standard set is an MCQ, which can assess application of knowledge (Miller’s level ‘knows how’) at best. However, criterion test was a workplace-based performance test, which assesses actual performance (Miller’s level of ‘does’). A candidate who scores well (or otherwise; i.e. poorly) in a ‘knows how’ test need not necessarily score similarly in a ‘does’ level due to the different levels (and constructs) of assessment. This is an inherent drawback of this study.
b The criterion test also had a pass mark of 4 out of 8. This was an arbitrary pass mark. It is not entirely scientific to compare the pass/fail rates of a test that had an arbitrary standard with the pass/fail rates of a test that was properly standard set.
c In the bookmark method, the standard setters were offered the pass mark that they set and the pass/fail rates at the end of each round, whereas in the Angoff method this information was not offered to the standard setters. This may be the reason why the bookmark method performed better in this study.
d In the bookmark method, we applied a 50% probability, and the results were based on that. If a probability of 67% was applied, the acceptance rate would have been different.
e Although the agreement of the panellists was compared between the two standard setting processes, it is not the panellist agreement (both intra-panellist and inter-panellist) that matters. It is the accuracy and appropriateness of the set standard that matters. A standard setting process could have perfect panellist agreement, but the standard set could be totally inaccurate, inappropriate and unacceptable. Hence, these agreement comparisons should be considered in this light.
f There was an unequal number of panellists in the two standard setting methods (11 vs. 12). This was due to the resignation of one of the experts. If the number of experts in both groups was the same, the comparison of the two methods would have been more acceptable. However, due to administrative problems and coordination issues with the panellists, the study was conducted using 12 and 11 experts.

## Conclusions

It is recommended that the future interested researchers compare the performance of these methods in different tests and with different samples. It is also recommended to planners of medical education and assessment centers prioritize bookmark in standard setting.

## Data Availability

The datasets during and/or analyzed during the current study available from the corresponding author on reasonable request.

## Abbreviation

IRT: Item response theory.

## References

[1] Zlatkin-Troitschanskaia O, Shavelson RJ, Pant HA (2018). Assessment of learning outcomes in higher education. Handbook on Measurement, Assessment, and Evaluation Higher Education.

[2] Hejri SM, Jalili M (2014). Standard setting in medical education: fundamental concepts and emerging challenges. Med J Islam Repub Iran.

[3] Aviso KB, Lucas RI, Tapia JF, Promentilla MA, Tan RR (2018). Identifying key factors to learning process systems engineering and process integration through DEMATEL. Chem Eng Trans.

[4] Çetin S, Gelbal S (2013). A comparison of bookmark and Angoff standard setting methods. Educ Sci Theory Pract.

[5] Kane M (1994). Validating the performance standards associated with passing scores. Rev Educ Res.

[6] Cusimano MD (1996). Standard setting in medical education. Acad Med.

[7] Elfaki OA, Salih KM (2015). Comparison of two standard setting methods in a medical students MCQs exam in internal medicine. Am J Med Med Sci.

[8] Cizek GJ, Bunch MB. Standard setting: a guide to establishing and evaluating performance standards on tests: SAGE Publications Ltd; 2007.

[9] Liu M, Liu KM (2008). Setting pass scores for clinical skills assessment. Kaohsiung J Med Sci.

[10] Jalili M, Mortazhejri S (2012). Standard setting for objective structured clinical exam using four methods: pre-fixed score, Angoff, borderline regression and Cohen’s. Strides Dev Med Educ.

[11] Mortaz Hejri S, Jalili M, Labaf A (2012). Setting standard threshold scores for an objective structured clinical examination using Angoff method and assessing the impact of reality Chacking and discussion on actual scores. Iran J Med Educ.

[12] Angoff W (1996). Scales, norms, and equivalent scores. Educational Measurement: Theories and applications.

[13] Mitzel HC, Lewis DM, Patz RJ, Green DR (2001). The bookmark procedure: psychological perspectives. Setting performance standards: Concepts, methods, and perspectives.

[14] Boursicot K (2006). Setting standards in a professional higher education course: defining the concept of the minimally competent student in performance-based assessment at the level of graduation from medical school. High Educ Q.

[15] Talente G, Haist SA, Wilson JF (2003). A model for setting performance standards for standardized patient examinations. Eval Health Prof.

[16] Norcini JJ (2003). Setting standards on educational tests. Med Educ.

[17] Kilminster S, Roberts T (2004). Standard setting for OSCEs: trial of borderline approach. Adv Health Sci Educ.

[18] Lin J (2006). The bookmark procedure for setting cut-scores and finalizing performance standards: strengths and weaknesses. Alberta J Educ Res.

[19] Cizek GJ, Bunch MB, Koons H (2004). Setting performance standards: Contemporary methods. Educational measurement: issues and practice.

[20] Lewis DM, Mitzel HC, Mercado RL, Schulz EM (2012). The bookmark standard setting procedure. Setting performance standards: Foundations, methods, and innovations.

[21] Hsieh M (2013). Comparing yes/no Angoff and bookmark standard setting methods in the context of English assessment. Lang Assess Q.

[22] Buckendahl CW, Smith RW, Impara JC, Plake BS (2002). A comparison of Angoff and bookmark standard setting methods. J Educ Meas.

[23] Reckase MD (2006). A conceptual framework for a psychometric theory for standard setting with examples of its use for evaluating the functioning of two standard setting methods. Educ Meas Issues Pract.

[24] Olsen JB, Smith R (2008). Cross validating modified Angoff and bookmark standard setting for a home inspection certification.

[25] Schulz EM (2006). Commentary: a response to Reckase's conceptual framework and examples for evaluating standard setting methods. Educ Meas Issues Pract.

[26] Hambleton RK, Pitoniak MJ (2006). Setting performance standards. Educ Meas.

[27] Plake BS (2008). Standard setters: stand up and take a stand!. Educ Meas Issues Pract.

[28] Cizek GJ, Downing SM, Haladyna TM (2006). Standard setting. Handbook of test development.

[29] Chong L, Taylor S, Haywood M, Adelstein BA, Shulruf B. The sights and insights of examiners in objective structured clinical examinations. J Educ Eval Health Prof. 2017;14. 10.3352/jeehp.2017.14.34 PMC5801428 29278906

[30] Yudkowsky R, Downing SM, Popescu M (2008). Setting standards for performance tests: a pilot study of a three-level Angoff method. Acad Med.

[31] Trevethan R (2017). Sensitivity, specificity, and predictive values: foundations, pliabilities, and pitfalls in research and practice. Front Public Health.

[32] Landis JR, Koch GG (1977). An application of hierarchical kappa-type statistics in the assessment of majority agreement among multiple observers. Biometrics..

[33] Krippendorff K (1980). Validity in content analysis.

[34] Steiger JH (1980). Tests for comparing elements of a correlation matrix. Psychol Bull.

[35] Yim M (2018). Comparison of results between modified-Angoff and bookmark methods for estimating cut score of the Korean medical licensing examination. Korean J Med Educ.

[36] Meskauskas JA (1986). Setting standards for credentialing examinations: an update. Eval Health Prof.

[37] Cizek GJ (2001). Setting performance standards.

[38] Lypson ML, Downing SM, Gruppen LD, Yudkowsky R (2013). Applying the bookmark method to medical education: standard setting for an aseptic technique station. Med Teach.

[39] Tiffin-Richards SP, Anand Pant H, Köller O (2013). Setting standards for English foreign language assessment: methodology, validation, and a degree of arbitrariness. Educ Meas Issues Pract.

[40] Pant HA, Rupp AA, Tiffin-Richards SP, Köller O (2009). Validity issues in standard-setting studies. Stud Educ Eval.

[41] Kampa N, Wagner H, Köller O (2019). The standard setting process: validating interpretations of stakeholders. Large Scale Assess Educ.

[42] Park J, Ahn DS, Yim MK, Lee J. Comparison of standard-setting methods for the Korean radiological technologist licensing examination: Angoff, Ebel, bookmark, and Hofstee. J Educ Eval Health Prof. 2018;15. 10.3352/jeehp.2018.15.32 PMC6380908 30586956

